# A scoping review of librarian involvement in competency-based medical education

**DOI:** 10.5195/jmla.2025.1965

**Published:** 2025-01-14

**Authors:** John W. Cyrus, Laura Zeigen, Molly Knapp, Amy E. Blevins, Brandon Patterson

**Affiliations:** 1 cyrusjw@vcu.edu, Research and Education Librarian, Health Sciences Library, Virginia Commonwealth University, Richmond, VA; 2 zeigenl@ohsu.edu, Health Sciences Education and Research Librarian, Oregon Health & Science University Library, Oregon Health & Science University, Portland, OR; 3 molly.knapp@utah.edu, Training Development Manager, Network of the National Library of Medicine National Training Office, Spencer S. Eccles Health Sciences Library, Salt Lake City, UT; 4 aeblevin@iu.edu, Associate Director for Public Services, Ruth Lilly Medical Library, Indiana University School of Medicine, Indianapolis, IN; 5 b.patterson@utah.edu, Technology Engagement Librarian, Spencer S. Eccles Health Sciences Library, University of Utah, Salt Lake City, UT

**Keywords:** Competency-Based Education, CBME, Evidence-Based Medicine, EBM, Problem-based learning, case-based learning, entrustable professional activities, self-regulated learning, lifelong learning, librarians, libraries, Instruction, education, learning, curriculum, training, undergraduate medical education

## Abstract

**Objective:**

A scoping review was undertaken to understand the extent of literature on librarian involvement in competency-based medical education (CBME).

**Methods:**

We followed Joanna Briggs Institute methodology and PRISMA-ScR reporting guidelines. A search of peer-reviewed literature was conducted on December 31, 2022, in Medline, Embase, ERIC, CINAHL Complete, SCOPUS, LISS, LLIS, and LISTA. Studies were included if they described librarian involvement in the planning, delivery, or assessment of CBME in an LCME-accredited medical school and were published in English. Outcomes included characteristics of the inventions (duration, librarian role, content covered) and of the outcomes and measures (level on Kirkpatrick Model of Training Evaluation, direction of findings, measure used).

**Results:**

Fifty studies were included of 11,051 screened: 46 empirical studies or program evaluations and four literature reviews. Studies were published in eight journals with two-thirds published after 2010. Duration of the intervention ranged from 30 minutes to a semester long. Librarians served as collaborators, leaders, curriculum designers, and evaluators. Studies primarily covered asking clinical questions and finding information and most often assessed reaction or learning outcomes.

**Conclusions:**

A solid base of literature on librarian involvement in CBME exists; however, few studies measure user behavior or use validated outcomes measures. When librarians are communicating their value to stakeholders, having evidence for the contributions of librarians is essential. Existing publications may not capture the extent of work done in this area. Additional research is needed to quantify the impact of librarian involvement in competency-based medical education.

## INTRODUCTION

The Association of Academic Health Science Libraries (AAHSL) formed the Competency-based Medical Education (CBME) Task Force on March 3, 2016, in order to identify the nature and depth of AAHSL Libraries' participation in the changes taking place in medical curricula highlighted by the adoption of Core Entrustable Professional Activities (Core EPAs). Competency-based medical education “is an outcomes-based approach to the design, implementation, and evaluation of education programs and to the assessment of learners across the continuum that uses competencies or observable abilities” [1]. Core EPAs, published in 2014 by the Association of American Medical Colleges (AAMC), provide a structure by which to measure the 13 basic competencies required by the Accreditation Council for Graduate Medical Education (ACGME) for residents going into their first day of residency. The EPAs represent the skills residents should learn in their undergraduate medical education. In particular, the EPAs include developing a well-formed clinical question to retrieve evidence to support clinical decision-making (EPA 7) and collaborating as part of an interprofessional team (EPA 9) [2]. Both of these are areas in which librarians have a vested interest and participate in the educational process of moving learners from pre-entrustable to entrustable. Thus, it is of critical importance to librarians working in medical education to understand where these competencies are being assessed. The work of the original AAHSL CBME task force resulted in the mapping of EPAs to the Association of College Research Libraries (ACRL) Information Literacy Framework and a survey of librarian involvement with EPA 7 that was later published in BMC Medical Education[3]. In August of 2019, a new AAHSL taskforce was established and charged with implementing competency-based medical education (CBME) taskforce recommendations. One of the goals of the new taskforce was to “create a collection of case studies, vignettes, best practice stories, or other representations demonstrating the beneficial roles and positive impacts of librarian engagement in competency-based medical education (CBME).”

The new task force referred to the work of the previous task force and examined relevant literature to guide their work. In their 2012 review, Dorsch and Perry found that while there were numerous studies published on the topic of librarian involvement in medical education, “gaps in the literature suggest a need for longitudinal follow-up and multicentered studies to validate the findings of the literature to date”[4]. A scoping review was selected for this research as the methodology lends itself both to the mapping of an area of research and the identification of gaps in existing research [5]. This scoping review seeks to understand the current state of librarian involvement in CBME and provide demonstrable evidence of the value of engaging in this work to both librarians and medical education stakeholders. Specifically, the review sought to answer what roles librarians play in supporting CBME, how interventions involving librarians are designed, which outcomes have been used to measure the impact of librarian work in CBME, and whether or not there is evidence that any of these outcomes affect clinical competence?

## METHODS

We performed a scoping review of published literature on librarian involvement in competency-based medical education in accordance with guidance from the Joanna Briggs Institute (JBI) Manual for Evidence Synthesis [6] and reported following the PRISM-ScR guidelines [7]. The protocol for this review is available through the Open Science Framework (https://osf.io/gcy4e).

The authors used the Association of American Medical Colleges' definition of CBME as “an outcomes-based approach to the design, implementation, and evaluation of education programs and to the assessment of learners across the continuum that uses competencies or observable abilities”[8]. In order to operationalize this definition for this review, the following concepts were included to describe content falling under the umbrella of CBME: entrustable professional activities (EPAs), self-directed learning (SDL), evidence-based medicine (EBM), interprofessional education (IPE), quality improvement, systems-based practice, health systems science, health services research, translational science, shared decision making, case-based learning, and problem-based learning.

The research team conducted searches in the following databases: Medline (Ovid), Embase (Ovid), ERIC (EBSCO), CINAHL Complete (EBSCO), SCOPUS (Elsevier), and Library & Information Science Source (LISS)/Library Literature & Information Science (LLIS)/Library, Information Science & Technology Abstracts (LISTA) via EBSCO. No multi-database searching was conducted. Each database was searched individually. An initial search was run on April 14, 2021, and an updated search was run to include articles published up to December 31, 2022. To be inclusive, controlled vocabulary terms and keywords for the concepts of competency-based medical education, critical thinking, evidence-based practice, and libraries/librarians were used. The concept “libraries/librarians” was specifically added since, without this, the search might return a body of results comprised of all the literature about CBME, not just the subsection mentioning librarians and libraries in the context of CBME. No filters for study type, date, or language were used. The search results were imported into Covidence systematic review management software (https://www.covidence.org/). Duplicate records were removed using Covidence. Full search strategies are included as Supplementary Material.

All screening took place in Covidence in two phases: title/abstract and full-text. Selection was conducted independently with two reviewers screening each study. Conflicts were resolved by consensus among the entire team. Eligibility criteria were established a priori. To be included in the review, papers had to describe librarian involvement in the planning, delivery, and/or assessment of competency-based medical instruction or educational intervention in undergraduate medical education (UME), the phase of medical education that confers the Doctor of Medicine (MD) degree. Additionally, studies need to be conducted in Liaison Committee on Medical Education (LCME) accredited medical schools located in the United States. The LCME is the accrediting body for education programs in the United States leading to an MD degree. Studies that were not published or available in the English language were excluded.

Following the process for charting described in Arksey & O'Malley [5], we extracted the following variables from each study into a spreadsheet generated using Google Forms: author name, date of publication, the title of the journal, the competency domain(s) assessed (based on EPAs where librarians self-identified involvement), and whether or not the outcomes addressed clinical competence. The components of EPA 7, which includes elements of EBM, were further mapped to four of the five A's of the EBM cycle. The competencies we assessed included:

- EPA 7 - Ask: Developing a well-formed, focused clinical question
- EPA 7 - Acquire: Awareness and skills in using information technology to access accurate and reliable medical information
- EPA 7 - Appraise: Skills in appraising sources, content, and applicability of evidence
- EPA 7 - Apply: Apply findings to individuals or populations, communicate findings to patient and team, reflecting on process
- EPA 9 - Identify team member roles and responsibilities and seek help other members of the team to optimize health care
- EPA 9 - Include team members, listen attentively, adjust communication content and style to align with team-member needs
- EPA 9 - Establish and maintain a climate of mutual respect, prioritize team needs over personal needs.

For empirical studies, defined as quantitative studies for this review, we extracted the dates of data collection, study aim, location of research, name of institution where research was conducted, population, intervention/exposure, duration of intervention/exposure, and librarian role in curriculum. The librarian roles in the curriculum were defined by the authors as follows based on the synthesis of existing literature: collaborator (librarian is not the instigator but involved in the teaching), curriculum designer (primarily involved in designing the curriculum), leader (instigator of curriculum or session), or evaluator (directly involved in the evaluation of student skills and knowledge gained through the curriculum) [4, 9, 10, 11]. The purpose of the study (program/curriculum evaluation, course/class evaluation, program/curriculum/course development, curriculum review/mapping), study design, direction of findings by outcome (positive, no change, negative, not reported), and the measure used for outcomes assessment were also extracted. Study outcomes were categorized by Kirkpatrick Model level [12]. The levels of this model, which is used to conceptualize how training is evaluated, includes reaction (learner reaction to and thoughts about their training experience), learning (learner change in knowledge from baseline as a result of the training), behavior (observable, measurable, repeatable behavior that the learner can demonstrate), and results (the tangible results of the training, such as improved patient outcomes). For evidence synthesis studies, which included both narrative reviews and more formal methodologies like systematic reviews, we collected the study aim/question(s), population/setting of interest, number and names of databases searched, date of last search, review design (literature, systematic, meta-analysis, scoping review, etc.), number of studies included, and the findings related to aim/research question (positive, no change, negative). The data extraction form was piloted with the entire group. Two reviewers extracted data from each study with a third reviewer to check the data and resolve conflicts.

We used descriptive statistics to describe the extent, nature, and distribution of the studies included in the review. In addition, we analyzed data related to publication dates and journals for all included studies. Studies were categorized by the characteristics of the interventions and by the levels of outcomes and how the outcomes were measured. Risk of bias assessment was not conducted for this scoping review as it was deemed not to provide useful information relevant to the research questions addressed by this review.

## RESULTS

**Figure 1:**
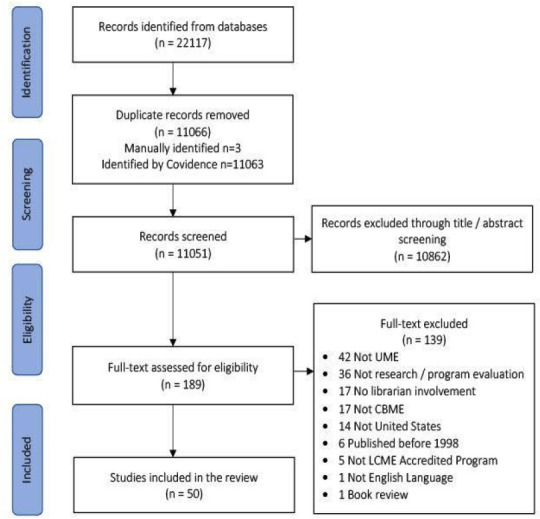
PRISMA Flow Diagram

Of the 11,051 studies screened for inclusion, 50 were included (Figure 1 PRISMA Flow Diagram). Forty-six articles were empirical research or program evaluation and four were some form of evidence synthesis. The articles were published in eight journals, including Academic Medicine (5), BMC Medical Education (1), BMJ Evidence Based Medicine (2), Health Libraries Review (1), Journal of the American Medical Informatics Association (1), Journal of the Medical Library Association or the Bulletin of the Medical Library Association (15), MedEdPORTAL (1), and Medical Reference Services Quarterly (24) between 1996 and 2022.

### Characteristics of the Interventions

The teaching strategies employed as part of the intervention varied, and generally included didactic lecture followed by individual or small group work. Content and structure were inconsistent. All teaching strategies reported positive outcomes. Teaching strategies included in person didactic lecture, online learning modules, pre-recorded lectures, PubMed practice searches, clinical case worksheets, presentations, small group work, and problem-based learning (see Table 1 Characteristics of interventions from reviewed studies).

**Table 1:** Characteristics of interventions from reviewed studies

Study ID	Population	Intervention	Duration of Intervention	Librarian Role (Leader, Collaborator, Curriculum Designer, Evaluator)	Domains Covered by Intervention*
Abate et al., 2011 [13]	1st year medical students	Four didactic sessions on resources, searching, and evidence-based medicine.	90 minutes x 4	Leader, Curriculum Designer	EPA 7-Acquire
Adams, 2015 [14]	1st year medical students	Course	Two weeks	A11 of the above	EPA 7-Ask
Aronoff et al., 2017 [15]	Students from nine health professions across two institutions (medical, dental, pharmacy, nursing, occupational therapy, physical therapy, social work, speech language path, dietetics)	Two online learning modules (“Intro to EBP” and “; Finding Evidence in PubMed”) followed by facilitated in-person small group case-based learning experience.	Two hours (1 hour per module)	Collaborator, Curriculum Designer, Evaluator	EPA 7-Acquire
Blake et al., 2018 [16]	1st and 2nd year medical students	Pre-recorded evidence-based medicine modules (Interviewing a standardized patient, Practice reaching a diagnosis, Practice searching PubMed and point of care tools for evidence)	A single 4-hour in-person session plus 3 hours of pre-recorded lectures	Collaborator	EPA 7-Ask
Blanco et al., 2014 [17]	Deans from AAMC medical schools	A cross-sectional survey.	n/a	Collaborator	EPA 7-Ask, EPA 7-Acquire, EPA 7-Appraise
Blumenthal et al., 2005 [18]	1st year medical students and 3rd year medical students on their family medicine rotation.	1st Year Students: large group sessions led by faculty-librarian team with student presentations, 3rd Year: 2-hour small group led by faculty-librarian team with students completing an EBM clinical case worksheet.	1st Year: not reported, 3rd Year: 2 hours	Collaborator	EPA 7-Ask, EPA 7-Acquire, EPA 7-Appraise, EPA 7-Apply
Brahmi et al., 1999 [19]	4th year medical students	Five two-hour sessions taught across one week (2 hours each day) on EBM, searching MEDLINE and Cochrane, and critical appraisal of research.	1 week: 2 hours a day for 5 days	Collaborator	EPA 7-Ask, EPA 7-Acquire, EPA 7-Appraise, EPA 7-Apply
Brown and Nelson, 2003 [20]	1st and 2nd year medical students	Longitudinal instruction in constructing clinical questions, searching skills, and library resources.	Multiple sessions over two years	Collaborator, Evaluator	EPA 7-Ask, EPA 7-Acquire, EPA 7-Appraise, EPA 7-Apply
Burrows and Tylman, 1999 [21]	3rd year medical students	Evaluation of literature searches before and after information resources and searching skills training.	1996-1998	Evaluator	EPA 7-Ask, EPA 7-Acquire, EPA 7-Appraise
Butera et al., 2014 [22]	1st year medical students	A combination of case-based scenarios and web-based information resources tailored to the assignment with direct librarian support for student research questions embedded into the course.	Semester length course	Collaborator, Curriculum Designer	EPA 7-Ask, EPA 7-Acquire
Cyrus et al., 2013 [23]	3rd and 4th year medical students	Described as a “selective” comprising two sessions: a library session on database searching and statistical concepts, a session on critical appraisal of preselected articles to emphasize statistics and research design, and a session on critical appraisal of articles submitted by students and re-emphasis of statistical concepts.	2 or 3 sessions	Collaborator	EPA 7-Ask, EPA 7-Acquire, EPA 7-Appraise
Dorsch et al., 2004 [24]	3rd year medical students	An evidence-based medicine seminar series of in-person group sessions	8 one-hour seminars during a 12-week internal medicine clinical rotation.	Collaborator, Evaluator	EPA 9-Identify
Earl, 1996 [25]	1st year medical students	A problem-based learning case and group work.	One-hour group work during class time.	A11 of the above	EPA 7-Ask, EPA 7-Acquire, EPA 7-Appraise
Eldredge et al., 1998 [26]	Librarians	Program description of a School of Medicine curriculum reform.	Not reported	Collaborator, Curriculum Designer	EPA 7-Ask, EPA 7-Acquire, EPA 7-Appraise, EPA 7-Apply
Eldredge et al., 2021 [27]	1st year medical students	A pre-post-design with the intervention consisting of a lecture on question formulation, a case vignette, and practice formulating clinical questions from the vignette.	Single session (duration not reported), including a 25-minute lecture from a librarian.	A11 of the above	EPA 7-Ask, EPA 7-Acquire
Gagliardi et al., 2012 [28]	3rd year medical students	A combination of large group lecture and case-based learning team taught by librarians and diverse clinical faculty.	Six two-hour sessions over six consecutive weeks	A11 of the above	Not reported
Gaines et al., 2018 [29]	1st and 2nd year medical students	Small group learning with librarian as the facilitator covering evidence-based medicine basics, clinical questions, searching, and matching library resources to the question.	1 or 3 weeks	A11 of the above	EPA 7-Ask
Getselman and White, 2011 [30]	1st year medical students.	A preassessment followed by a 30-minute lecture and a 90-minute active review of the concepts.	2 hours	A11 of the above	EPA 7-Ask, EPA 7-Acquire, EPA 7-Appraise, EPA 7-Apply
Geyer and Irish, 2008 [31]	1st, 2nd, 3rd, and 4th year medical students.	A combination of web-based module consisting of tutorials and assessments, large group lecture, case-based learning, and individual assistance.	Session length varied by year.	Collaborator, Curriculum Designer, Evaluator	EPA 7-Ask, EPA 7-Acquire
Gibson and Silverberg, 2000 [32]	1st year medical students.	Seven hours of instruction over two sessions covering computer operating systems, basic computer literacy, and searching MEDLINE and the library catalog.	7 hours over 2 sessions	All of the above	EPA 7-Acquire
Gruppen et al., 2005 [33]	4th year medical students.	10 sessions of lecture and discussion on types of research literature (e.g., therapy, diagnosis, guidelines). Individual student work to generate 5 clinical questions, find evidence to answer the question, and appraise its validity throughout the elective. Pre-post design was used to assess student knowledge and skills.	4 weeks including 10 90-minute lectures	Collaborator, Evaluator	EPA 7-Ask, EPA 7-Acquire, EPA 7-Appraise, EPA 7-Apply
Haley, 2019 [34]	80 total participants including 13 medical students	A single 1-hour interprofessional group book discussion facilitated by a librarian and faculty member. Pre-post survey delivered to measure interprofessional education knowledge and attitudes.	1 hour	Collaborator	EPA 7-Acquire
Hersh et al., 2002 [35]	66 total participants including 45 4th year medical students.	A large group lecture session on MEDLINE and overview of evidence-based medicine followed by two hands-on sessions applying content from the lectures 2–4 weeks after the lecture.	30 minutes large group lecture plus two 2-hour hands-on sessions.	Collaborator	EPA 7-Ask, EPA 7-Acquire
Kaplowitz and Wilkerson, 2022 [36]	1st year medical students.	A guided tour of the library, a small group review of library resources, and a large group discussion of resources.	45 minutes	Curriculum Designer	EPA 9-Identify, EPA 9-Include, EPA 9-Establish
Kaufman et al., 1999 [37]	1st year medical students	A single introductory large-group lecture and discussion followed by four modules on evidence-based medicine resources and skills and a final project.	10 weeks	Collaborator, Curriculum Designer, Evaluator	EPA 7-Ask, EPA 7-Acquire
Lawrence and Levy, 2004 [38]	571 participants including 401 medical students.	A single workshop consisting of assessed via pre-/post-test of MEDLINE searching skills.	One session (duration not reported)	A11 of the above	EPA 7-Acquire
MacEachern et al., 2012 [39]	1st, 2nd, 3rd, and 4th year medical students.	A combination of lecture, case-based learning, and discussion covering various topics: Information resources (background, clinical), database searching skills.	Duration of sessions varied by year: 3 hours (1st year), 2 hours (2nd year), 2+ hours (3rd year), estimated 1 hour (4th year).	Collaborator	EPA 7-Ask, EPA 7-Acquire, EPA 7-Appraise, EPA 7-Apply
Menard et al., 2021 [40]	3rd year medical students in their internal medicine clerkship.	Content included information resources, searching, and critical appraisal skills followed by an evidence-based medicine assignment. Intervention varied by class year but specific education strategies and methods were not reported.	14 hours instruction at the beginning of the first 2 weeks of medical school with evidence-based medicine assignments taking place during the second year.	Collaborator, Curriculum Designer, Evaluator	EPA 7-Ask, EPA 7-Acquire EPA 7-Appraise
Minuti et al., 2018 [41]	1st and 2nd year medical students.	An interactive online tutorial covering clinical questions, searching, and information resources and a classroom session consisting of lecture and small group work.	1–2 hours	Collaborator, Curriculum Designer, Evaluator	EPA 7-Ask, EPA 7-Acquire,
Morley and Hendrix, 2012 [42]	students participating in elective course 2nd and 3rd year medical students	Combination of lecture, individual hands-on work, and group discussion.	A semester-long course of 7.5 hours	Curriculum Designer, Evaluator	EPA 7-Ask, EPA 7-Acquire, EPA 7-Appraise, EPA 7-Apply
Muellenbach et al., 2018 [43]	1st year medical students	2 Ripped classroom (pre-work, discussion, case-based learning) evidence-based medicine sessions covering an overview of EBM, information resources, clinical questions, and searching skills.	2 one-hour sessions	Collaborator, Curriculum Designer	EPA 7-Ask, EPA 7-Acquire, EPA 7-Appraise, EPA 7-Apply
Nelson, 2018 [44]	3rd year medical students	Two online modules course covering a review of evidence-based medicine concepts, skills, and point of care resources.	Duration not reported	A11 of the above	EPA 7-Acquire, EPA 7-Appraise
Nevius et al., 2018 [45]	US and Canadian libraries and LCME-accredited medical schools.	A twenty-eight-question survey with a mixture of qualitative and quantitative questions.	Not reported	A11 of the above	EPA 7-Ask, EPA 7-Acquire, EPA 7-Appraise
Nicholson et al., 2019 [3]	US and Canadian health sciences libraries.	A survey assessing the extent of librarian are involved in teaching EPA 7 content, including how it is being taught, assessed, the depth of content being taught,	Not reported	A11 of the above	EPA 7-Ask, EPA 7-Acquire
O'Dwyer and Kerns, 2011 [46]	1st and 2nd year medical students.	Problem-based learning sessions on clinical questions, and appraising research.	12 weeks	Curriculum Designer, Evaluator	Not reported
Skhal, 2008 [47]	3rd year medical students.	Orientation session on information resources for each clinical rotation in Pediatrics, and Internal Medicine totaling 28 sessions annually followed by case-based assignment assessed by pre-post test		Leader, Collaborator, Curriculum Designer	EPA 7-Ask, EPA 7-Acquire, EPA 7-Appraise, EPA 7-Apply
Swanberg et al., 2017 [48]	2nd year medical students	An instructional session as part of a comprehensive evidence-based medicine course covering clinical questions, searching for evidence, and appraising research.	Three-hour session consisting of a50-minute lecture followed by a mandatory 2-hour interactive lab.	Leader, Collaborator, Curriculum Designer, Evaluator	EPA 7-Ask, EPA 7-Appraise
Tagge, 2018 [49]	1st year medical students	Case-based learning covering all aspects of the evidence-based medicine process.	One 2-hour session	Leader, Collaborator, Curriculum Designer, Evaluator	EPA 7-Ask, EPA 7-Acquire, EPA 7-Appraise, EPA 7-Apply
Thomas et al., 2020 [50]	1st and 2nd year medical students	Didactic on evidence-based medicine basics and searching PubMed followed by a small group hands-on activity.	90 minutes to 2 hours	Collaborator, Curriculum Designer, Evaluator	EPA 7-Ask, EPA 7-Acquire, EPA 7-Appraise, EPA 7-Apply
Turner et al., 2017 [51]	1st year medical students	A single session on searching in PubMed.	A single 70-minute session	Leader, Collaborator, Curriculum Designer, Evaluator	EPA 7-Ask, EPA 7-Acquire, EPA 7-Appraise, EPA 7-Apply
Wallach et al., 2002 [52]	1st year medical students	A mix of lecture, small group work covering finding evidence and appraising research.	Not reported	Collaborator	EPA 7-Ask, EPA 7-Acquire
Whipple et al., 2009 [53]	1st year medical students	Lecture covering background questions, using information resources to answer them, followed by case study small group exercise.	1 hour	Curriculum Designer	EPA 7-Ask, EPA 7-Acquire, EPA 7-Appraise, EPA 7-Apply
Wiecha et al., 2002 [54]	3rd year medical students	Online modules covering finding evidence, appraising research, and applying evidence to a patient.	6 weeks	Curriculum Designer, Evaluator	EPA 7-Acquire, EPA 7-Appraise
Wong and Ren, 2022 [55]	1st year medical students	A single session on library resources, advanced search strategies, and critical appraisal.	90 minutes	Leader	EPA 7-Acquire
Wrosch et al., 1998 [56]	1st year medical students	A lecture on searching in MEDLINE followed by small group work answering an assigned clinical question, and appraising an article.	A single two-hour session.	Collaborator, Curriculum Designer	EPA 7-Acquire, EPA 7-Appraise, EPA 7-Apply
Zeigen and Hamilton, 2021 [57]	1st year medical students	A lecture on clinical questions and literature searching with mandatory follow-up consultation.	A single one-hour session plus mandatory consultation with a librarian.	Collaborator	EPA 7-Acquire, EPA 7-Appraise

* Domains covered are Entrustable Professional Activities (EPAs) and include stages of developing a well-formed clinical question to retrieve evidence to support clinical decision making (EPA 7: Ask, Acquire, Appraise, Apply) and collaborating as part of an interprofessional team (EPA 9: Identify, Include, Establish).

The duration of the intervention also varied. On one end there was a 30-minute lecture on PubMed/MEDLINE and on the other end was teaching concepts longitudinally throughout an entire semester or over several years. While formal statistical analysis was not conducted to test the relationship, the duration of the intervention did not appear to correlate with positive results. The studies without specifically reported positive results included a semester-long class and multiple 60 to 120-minute one-shot interventions. Librarians were active in every role that we used as a category with the most common role being Collaborator (36) followed by Curriculum Designer (30), Leader (16), and Evaluator (10) (see Table 2 Librarian roles from review studies).

**Table 2:** Librarian Roles from Reviewed Studies

	Librarian Role			
	Leader	Collaborator	Curriculum Designer	Evaluator
Study ID				
Abate et al., 2011 [13]	X	-	X	-
Adams, 2015 [14]	X	X	X	X
Aronoff et al., 2017 [15]	-	X	X	X
Blake et al., 2018 [16]	-	X	-	-
Blanco et al., 2014 [17]	-	X	-	-
Blumenthal et al., 2005 [18]	-	X	-	-
Brahmi et al., 1999 [19]	-	X	-	-
Brown and Nelson, 2003 [20]	-	X	-	X
Burrows and Tylman, 1999 [21]	-	-	-	X
Butera et al., 2014 [22]	-	X	X	-
Cyrus et al., 2013 [23]	-	X	-	-
Dorsch et al., 2004 [24]	-	X	-	X
Earl, 1996 [25]	X	X	X	X
Eldredge et al., 1998 [26]	-	X	X	-
Eldredge et al., 2021 [27]	X	X	X	X
Gagliardi et al., 2012 [28]	X	X	X	X
Gaines et al., 2018 [29]	X	X	X	X
Getselman and White, 2011 [30]	X	X	X	X
Geyer and Irish, 2008 [31]	-	X	X	X
Gibson and Silverberg, 2000 [32]	X	X	X	X
Gruppen et al., 2005 [33]	-	X	-	X
Haley, 2019 [34]	-	X	-	-
Hersh et al., 2002 [35]	-	X	-	-
Kaplowitz and Wilkerson, 2022 [36]	-	-	X	-
Kaufman et al., 1999 [37]	-	X	X	X
Lawrence and Levy, 2004 [38]	X	X	X	X
MacEachem et al., 2012 [39]	-	X	-	-
Menard et al., 2021 [40]	-	X	X	X
Minuti et al., 2018 [41]	-	X	X	X
Morley and Hendrix, 2012 [42]	-	X	-	X
Muellenbach et al., 2018 [43]	-	X	X	-
Nelson, 2018 [44]	X	X	X	X
Nevius et al., 2018 [45]	X	X	X	X
Nicholson et al., 2019 [3]	X	X	X	X
O'Dwyer and Kerns, 2011 [46]	-	-	X	X
Skhal, 2008 [47]	X	X	X	-
Swanberg et al., 2017 [48]	X	X	X	X
Tagge, 2018 [49]	X	X	X	X
Thomas et al., 2020 [50]	-	X	X	X
Turner et al., 2017 [51]	X	X	X	X
Wallachet al., 2002 [52]	-	X	-	-
Whipple et al., 2009 [53]	-	-	X	-
Wiechaetal., 2002 [54]	-	-	X	X
Wong and Ren, 2022 [55]	X	-	-	-
Wrosch et al., 1998 [56]	-	X	X	-
Zeigen and Hamilton, 2021 [57]	-	X	-	-

The most common domains covered by interventions were EPA 7 Appraise (n=39, 78%), followed by EPA 7 Ask (n=32, 64%) and EPA 7 Acquire (n=29, 58%). Other domains were covered less extensively, including EPA 7 Advise (n=15, 30%) and all domains associated with EPA 9 (Team Roles (n=2, 4%), Mutual Respect (n=1, 2%), and Team Communication (n=1, 2%).

The majority of studies used unvalidated outcomes measures and there was little consistency among the outcomes that were assessed (see Table 3 Outcomes of interventions from reviewed Studies). Of the tools used to measure the effect of the interventions, three studies utilized a modified Fresno test and one used the Berlin questionnaire [15, 28, 40]. The Fresno test and Berlin questionnaire are two of a handful small number of validated scales that assess competence in evidence-based practice knowledge and skills [58, 59]. Seven studies used a pre-/post-intervention evaluation design, largely through anonymous/online surveys [23, 24, 33, 34, 38, 47, 50]. The remaining studies required students to synthesize or actively apply knowledge asking them to develop a case scenario and make a case or team presentation, perform in an objective structured clinical examination (OSCE) case study, create a patient-centered disease information resource sheet, answer questions that asked them to identify the highest quality of evidence in sources, or submit multiple MEDLINE search strategies that were then evaluated by librarians. Three studies used a rubric to evaluate assignments, but details on the composition or creation of the rubric were not given [31, 48]. Only two studies specifically mentioned giving formative feedback to learners [31, 36].

**Table 3:** Outcome interventions from Reviewed Studies

	Outcome Interventions			
	Reaction	Learning	Behavior	Results
Study ID				
Abate et al., 2011 [13]	Positive	-	-	-
Adams, 2015 [14]	Positive	Positive	-	-
Aronoff et al., 2017 [15]	Positive	Positive	-	-
Blake et al., 2018 [16]	Positive	-	-	-
Blanco et al., 2014 [17]	-	-	-	-
Blumenthal et al., 2005 [18]	Positive	-	-	-
Brahmi et al., 1999 [19]	Positive	-	-	-
Brown and Nelson, 2003 [20]	-	-	Positive	-
Burrows and Tylman, 1999 [21]	-	Negative	-	Negative
Butera et al., 2014 [22]	-	-	-	-
Cyrus et al., 2013 [23]	-	Positive	-	Positive
Dorsch et al., 2004 [24]	Positive	Positive	Positive	Positive
Earl, 1996 [25]	-	Positive	-	-
Eldredge et al., 1998 [26]	-	-	-	-
Eldredge et al., 2021 [27]	-	Positive	Positive	-
Gagliardi et al., 2012 [28]	Positive	Positive	Positive	-
Gaines et al., 2018 [29]	-	Positive	Positive	-
Getselman and White, 2011 [30]	Positive	-	-	-
Geyer and Irish, 2008 [31]	Positive	Positive	Positive	Positive
Gibson and Silverberg, 2000 [32]	Positive	Positive	-	-
Gruppen et al., 2005 [33]	-	Positive	Positive	-
Haley, 2019 [34]	Positive	Positive	-	-
Hersh et al., 2002 [35]	Positive	Positive	Positive	-
Kaplowitz and Wilkerson, 2022 [36]	Positive	-	-	-
Kaufman et al., 1999 [37]	Positive	Positive	Positive	-
Lawrence and Levy, 2004 [38]	Positive	Positive	-	-
MacEachem et al., 2012 [39]	-	-	-	-
Menard et al., 2021 [40]	Positive	Positive	Positive	-
Minuti et al., 2018 [41]	Positive	Positive	-	-
Morley and Hendrix, 2012 [42]	Positive	-	-	-
Muellenbach et al., 2018 [43]	Positive	-	-	-
Nelson, 2018 [44]	Positive	-	-	-
Nevius et al., 2018 [45]	-	-	-	-
Nicholson et al., 2019 [3]	-	-	-	-
O'Dwyer and Kerns, 2011 [46]	Positive	Positive	-	-
Skhal, 2008 [47]	Positive	Positive	-	-
Swanberg et al., 2017 [48]	Positive	Positive	-	-
Tagge, 2018 [49]	Positive	Positive	-	Positive
Thomas et al., 2020 [50]	Positive	Positive	-	-
Turner et al., 2017 [51]	Positive	-	-	-
Wallach et al., 2002 [52]	Positive	-	-	-
Whipple et al., 2009 [53]	Positive	Positive	-	-
Wiecha et al., 2002 [54]	Positive	Positive	-	-
Wong and Ren, 2022 [55]	-	Positive	-	-
Wrosch et al., 1998 [56]	-	-	-	-
Zeigen and Hamilton, 2021 [57]	-	-	-	-

Positive: findings found related to aim/research question provides positive and favorable results; Negative: findings found related to aim/research question provides negative and non-favorable results.

Study outcomes were categorized by Kirkpatrick Model level, which describes outcomes by the type of data that they collect and what that data conveys. The majority of studies assessed satisfaction and knowledge retention outcomes with 30 (65%) looking at reaction and 26 (57%) looking at knowledge outcomes. Fewer studies looked at outcomes that might transfer to clinical practice, such as the impact of an intervention on behaviors or how the interventions impact downstream results (learner, patient, clinical outcomes) with 11 (24%) looking at behavior and 6 (13%) looking at results as outcomes. More than 95% of studies reported positive outcomes; however, no study directly addressed the clinical competence of the learners.

### Characteristics of Evidence Syntheses

Four evidence syntheses articles explored various ways librarians involve themselves in CBME [4, 10–11, 60]. Out of 17 databases, the most commonly searched databases regardless of platform were MEDLINE (n=4), CINAHL (n=3), Embase (n=2), Web of Science (n=2), Scopus (n=2), ERIC (n=2), and PsycINFO (n=2). One article was a narrative review that reported methods but did not adhere to a specific methodological framework [4]. The remaining articles following scoping review [60] and systematic review methodologies [10, 11]. All evidence synthesis papers aimed to describe and assess instructional methods for teaching evidence-based practice concepts and skills, including searching, to health sciences or medical students. All syntheses reported results that trended positive but varied significantly from study to study. All studies also reported challenges in synthesizing evidence based on the diversity of interventions and outcomes measures, and a lack of standardized assessment tools. These studies also highlighted the disparate roles played by librarians in instruction, ranging from lecturer to curriculum designer, and the need to report detailed, standardized descriptions of educational interventions.

## DISCUSSION

This scoping review found that there is a strong base of literature on the involvement of librarians in competency-based medical education. Despite this, few studies included in this review assessed outcomes related to the application of knowledge or skills taught by a librarian or used validated measures to determine the effect of the intervention. The majority of studies reported generally positive outcomes related to reaction to the intervention or knowledge retention of the content. At the same time, outcomes related to behavior of the participants or outcomes related to the application of the skills or knowledge were rarely studied.

Similar to prior reviews [4, 9–11], this scoping review found that there was a high degree of variation in how the included studies were conducted. The teaching methods, duration, setting, and assessment methods varied from study to study, making comparisons between the existing evidence challenging. This study highlights the need for more standardized interventions and assessments, especially that which could result in the understanding of the librarian's role in ensuring clinical competence among learners. When authors are writing about CBME involvement, they should include detailed descriptions about their involvement and employ more rigorous research methods to allow others to draw conclusions about efficacy.

When librarians are communicating their value to internal and external stakeholders, having landmark studies with demonstrable evidence of the contributions of librarians is essential. While librarians are publishing articles related to their involvement in competency-based medical education, existing literature may not capture the extent of work done in this area. Additional research is needed to quantify the impact of librarian involvement in competency-based medical education.

## LIMITATIONS

As with any large-scale synthesis of evidence, decisions made during the design and search processes may introduce bias into the study. The decision to restrict eligibility to articles that were published or available in English and took place in LCME-accredited medical schools based in the United States potentially limited the pool of articles that could have informed our guiding questions. Additionally, hand searching of journals and conference abstracts was not conducted as part of this review due to lack of time.

## Data Availability

Data associated with this article, including Excel documentation spreadsheet, are on the Open Science Framework Site for this project (https://osf.io/gcy4e).

## SUPPLEMENTAL FILES

**Appendix A:** Search Strategies
