# Supplementary material for: A scoping review of librarian involvement in competency-based medical education
Source: J Med Libr Assoc. 2025 Jan 14;113(1):9–23. doi: 10.5195/jmla.2025.1965 (PMC11835034; doi:10.5195/jmla.2025.1965)
Supplement: Supplementary file 1 — Appendix A: Search Strategies [file jmla-113-1-9-s01.docx]

**Supplementary Material**

MEDLINE (Ovid) Search Strategy

| 1 | exp Quality Improvement/ or exp Metacognition/ or (quality improvement or self assessment or metacognition or scientific method or problem solving or critical judgment or critical thinking).mp. |
| --- | --- |
| 2 | Health Personnel/ or Allied Health Personnel/ or Faculty, Medical/ or Medical Staff/ or Nurses/ or Occupational Therapists/ or Pharmacists/ or Physical Therapists/ or Physicians/ or (health science or health sciences or medicine or medical or Anaesthesiologist* or anesthesiologist* or dentist* or dental* or doctor* or physician* or "nurse practitioner*" or nurse or clinician* or midwi* or pharmacist* or physiotherapist* or "occupational therapist*" or radiographer* or "medical personnel" or "medical professional*" or "medical worker*" or "medical provider*" or "medical staff" or "health personnel" or "healthcare personnel" or "health professional*" or "health care professional*" or "healthcare professional*" or "health worker*" or "health care worker*" or "healthcare worker*" or "health provider*" or "health care provider*" or "healthcare provider*" or "health staff" or "health care staff" or "healthcare staff" or Paramedic* or "health manager*" or "health care manager*" or "healthcare manager*" or "clinical officer*" or "community health aid*" or Physical therapy or physiotherapy or occupational therapy or rehabilitation therapy or osteopathy).mp. |
| 3 | 1 and 2 |
| 4 | exp Health Services Research/ or exp Translational Medical Research/ or exp Decision Making, Shared/ or (System based practice or Health systems science or Health services research or Translational research or Translational science or Shared decision making).mp. |
| 5 | 3 or 4 |
| 6 | exp curriculum/ or Learning/ or exp Education/ or (education or learning or curriculum or training).mp. |
| 7 | 5 and 6 |
| 8 | (Competency based medical education or Competency-based medical education or CBME or Entrustable professional activities or EPAs or Self-directed learning or self-regulated learning or IPE or Interprofessional education or inter-professional education or Case based learning or Problem based learning or lifelong learning or Translational medical education).mp. |
| 9 | exp Information Literacy/ or information literacy.mp. |
| 10 | 2 and 9 |
| 11 | 7 or 8 or 10 |
| 12 | (library or libraries or librarians or Informationist* or information specialist* or Information professional*).mp. or exp Librarians/ or exp Libraries/ |
| 13 | 11 and 12 |

Embase (Ovid) Search Strategy

| 1 | exp total quality management/ or exp Metacognition/ or (quality improvement or self assessment or metacognition or scientific method or problem solving or critical judgment or critical thinking).mp. |
| --- | --- |
| 2 | exp health care personnel/ or exp paramedical personnel/ or exp Medical Staff/ or exp Nurse/ or exp occupational therapist/ or exp Pharmacist/ or exp physiotherapist/ or exp Physician/ or (health science or health sciences or medicine or medical or Anaesthesiologist* or anesthesiologist* or dentist* or dental* or doctor* or physician* or "nurse practitioner*" or nurse or clinician* or midwi* or pharmacist* or physiotherapist* or "occupational therapist*" or radiographer* or "medical personnel" or "medical professional*" or "medical worker*" or "medical provider*" or "medical staff" or "health personnel" or "healthcare personnel" or "health professional*" or "health care professional*" or "healthcare professional*" or "health worker*" or "health care worker*" or "healthcare worker*" or "health provider*" or "health care provider*" or "healthcare provider*" or "health staff" or "health care staff" or "healthcare staff" or Paramedic* or "health manager*" or "health care manager*" or "healthcare manager*" or "clinical officer*" or "community health aid*" or Physical therapy or physiotherapy or occupational therapy or rehabilitation therapy or osteopathy).mp. |
| 3 | 1 and 2 |
| 4 | exp Health Services Research/ or exp translational research/ or exp shared decision making/ or (System based practice or Health systems science or Health services research or Translational research or Translational science or Shared decision making).mp. |
| 5 | 3 or 4 |
| 6 | exp curriculum/ or Learning/ or exp Education/ or (education or learning or curriculum or training).mp. |
| 7 | 5 and 6 |
| 8 | (Competency based medical education or Competency-based medical education or CBME or Entrustable professional activities or EPAs or Self-directed learning or self-regulated learning or IPE or Interprofessional education or inter-professional education or Case based learning or Problem based learning or lifelong learning or Translational medical education).mp. |
| 9 | exp Information Literacy/ or information literacy.mp. |
| 10 | 2 and 9 |
| 11 | 7 or 8 or 10 |
| 12 | (library or libraries or librarians or Informationist* or information specialist* or Information professional*).mp. or exp Librarian/ or exp Library/ |
| 13 | 11 and 12 |

ERIC (EBSCO) Search Strategy

| S1 | Quality Improvement or Metacognition or (quality improvement or self assessment or metacognition or scientific method or problem solving or critical judgment or critical thinking) |
| --- | --- |
| S2 | Health Personnel/ or Allied Health Personnel/ or Faculty, Medical/ or Medical Staff/ or Nurses/ or Occupational Therapists/ or Pharmacists/ or Physical Therapists/ or Physicians/ or (health science or health sciences or medicine or medical or Anaesthesiologist* or anesthesiologist* or dentist* or dental* or doctor* or physician* or "nurse practitioner*" or nurse or clinician* or midwi* or pharmacist* or physiotherapist* or "occupational therapist*" or radiographer* or "medical personnel" or "medical professional*" or "medical worker*" or "medical provider*" or "medical staff" or "health personnel" or "healthcare personnel" or "health professional*" or "health care professional*" or "healthcare professional*" or "health worker*" or "health care worker*" or "healthcare worker*" or "health provider*" or "health care provider*" or "healthcare provider*" or "health staff" or "health care staff" or "healthcare staff" or Paramedic* or "health manager*" or "health care manager*" or "healthcare manager*" or "clinical officer*" or "community health aid*" or Physical therapy or physiotherapy or occupational therapy or rehabilitation therapy or osteopathy) |
| S8 | S1 and S2 |
| S3 | Health Services Research/ or Translational Medical Research/ or Decision Making, Shared/ or (System based practice or Health systems science or Health services research or Translational research or Translational science or Shared decision making) |
| S9 | S3 or S8 |
| S4 | curriculum/ or Learning/ or Education/ or (education or learning or curriculum or training) |
| S10 | S4 and S9 |
| S5 | (Competency based medical education or Competency-based medical education or CBME or Entrustable professional activities or EPAs or Self-directed learning or self-regulated learning or IPE or Interprofessional education or inter-professional education or Case based learning or Problem based learning or lifelong learning or Translational medical education) |
| S6 | Information Literacy/ or information literacy |
| S11 | S2 and S6 |
| S12 | S5 or S10 or S11 |
| S7 | (library or libraries or librarians or Informationist* or information specialist* or Information professional*) or Librarians/ or Libraries/ |
| S13 | S7 and S12 |

CINAHL (EBSCO) Search Strategy

| S15 | S14  Narrow by Language: - english |
| --- | --- |
| S14 | S13  Limiters - Published Date: 19980101-20211231 (Updated to current date) |
| S13 | S11 AND S12 |
| S12 | library or libraries or librarians or Informationist* or information specialist* or Information professional* |
| S11 | S7 OR S8 OR S10 |
| S10 | S2 AND S9 |
| S9 | information literacy |
| S8 | Competency based medical education or Competency-based medical education or CBME or Entrustable professional activities or EPAs or Self-directed learning or self-regulated learning or IPE or Interprofessional education or inter-professional education or Case based learning or Problem based learning or lifelong learning or Translational medical education |
| S7 | S5 AND S6 |
| S6 | education or learning or curriculum or training |
| S5 | S3 OR S4 |
| S4 | System based practice or Health systems science or Health services research or Translational research or Translational science or Shared decision making |
| S3 | S1 AND S2 |
| S2 | health science or health sciences or medicine or medical or Anaesthesiologist* or anesthesiologist* or dentist* or dental* or doctor* or physician* or "nurse practitioner*" or nurse or clinician* or midwi* or pharmacist* or physiotherapist* or "occupational therapist*" or radiographer* or "medical personnel" or "medical professional*" or "medical worker*" or "medical provider*" or "medical staff" or "health personnel" or "healthcare personnel" or "health professional*" or "health care profession*" or "health worker*" or "health care worker*" or "healthcare worker*" or "health provider*" or "health care provider*" or "healthcare provider*" or "health staff" or "health care staff" or "healthcare staff" or Paramedic* or "health manager*" or "health care manager*" or "healthcare manager*" or "clinical officer*" or "community health aid*" or Physical therapy or physiotherapy or occupational therapy or rehabilitation therapy or osteopathy |
| S1 | quality improvement or self assessment or metacognition or scientific method or problem solving or critical judgment or critical thinking |

Library Literature & Information Science Index (H.W. Wilson) (EBSCO) Search Strategy

| S13 | S11 AND S12 |
| --- | --- |
| S12 | library or libraries or librarians or Informationist* or information specialist* or Information professional* |
| S11 | S7 OR S8 OR S10 |
| S10 | S2 AND S9 |
| S9 | information literacy |
| S8 | Competency based medical education or Competency-based medical education or CBME or Entrustable professional activities or EPAs or Self-directed learning or self-regulated learning or IPE or Interprofessional education or inter-professional education or Case based learning or Problem based learning or lifelong learning or Translational medical education |
| S7 | S5 AND S6 |
| S6 | education or learning or curriculum or training |
| S5 | S3 OR S4 |
| S4 | System based practice or Health systems science or Health services research or Translational research or Translational science or Shared decision making |
| S3 | S1 AND S2 |
| S2 | health science or health sciences or medicine or medical or Anaesthesiologist* or anesthesiologist* or dentist* or dental* or doctor* or physician* or "nurse practitioner*" or nurse or clinician* or midwi* or pharmacist* or physiotherapist* or "occupational therapist*" or radiographer* or "medical personnel" or "medical professional*" or "medical worker*" or "medical provider*" or "medical staff" or "health personnel" or "healthcare personnel" or "health professional*" or "health care profession ... |
| S1 | quality improvement or self assessment or metacognition or scientific method or problem solving or critical judgment or critical thinking |

Library and Information Science Source (EBSCO) Search Strategy

| S13 | S11 AND S12 |
| --- | --- |
| S12 | Librar* or Informationist* or “information specialist*” or “Information professional*” |
| S11 | S7 OR S8 OR S10 |
| S10 | S2 AND S9 |
| S9 | “information literacy” |
| S8 | “Competency based medical education” or “Competency-based medical education” or CBME or “Entrustable professional activities” or EPAs or “Self-directed learning” or “self-regulated learning” or IPE or “Interprofessional education” or “inter-professional education” or “Case based learning” or “Problem based learning” or “lifelong learning” or “Translational medical education” |
| S7 | S5 AND S6 |
| S6 | education or learning or curriculum or training or teaching |
| S5 | S3 OR S4 |
| S4 | “System* based practice” or “Health system* science” or “Health services research” or “Translational research” or “Translational science*” or “Shared decision making” |
| S3 | S1 AND S2 |
| S2 | “health science*” or medicine or medical or anaesthesiologist* or anesthesiologist* or dentist* or dental* or doctor* or physician* or "nurse practitioner*" or nurse or clinician* or midwi* or pharmacist* or physiotherap* or "occupational therap*" or radiographer* or "medical personnel" or "medical professional*" or "medical worker*" or "medical provider*" or "medical staff" or "health personnel" or "healthcare personnel" or "health professional*" or "health care professional*" or "healthcare professional*" or "health worker*" or "health care worker*" or "healthcare worker*" or "health provider*" or "health care provider*" or "healthcare provider*" or "health staff" or "health care staff" or "healthcare staff" or Paramedic* or "health manager*" or "health care manager*" or "healthcare manager*" or "clinical officer*" or "community health aid*" or “Physical therap*” or “rehabilitation therapy” or osteopath* |
| S1 | “quality improvement” or “self assessment*” or metacognition or “scientific method” or “problem solving” or “critical judgment” or “critical thinking” |

Library, Information Science & Technology Abstracts (EBSCO) Search Strategy

| S13 | S11 AND S12 |
| --- | --- |
| S12 | Librar* or Informationist* or “information specialist*” or “Information professional*” |
| S11 | S7 OR S8 OR S10 |
| S10 | S2 AND S9 |
| S9 | “information literacy” |
| S8 | “Competency based medical education” or “Competency-based medical education” or CBME or “Entrustable professional activities” or EPAs or “Self-directed learning” or “self-regulated learning” or IPE or “Interprofessional education” or “inter-professional education” or “Case based learning” or “Problem based learning” or “lifelong learning” or “Translational medical education” |
| S7 | S5 AND S6 |
| S6 | education or learning or curriculum or training or teaching |
| S5 | S3 OR S4 |
| S4 | “System* based practice” or “Health system* science” or “Health services research” or “Translational research” or “Translational science*” or “Shared decision making” |
| S3 | S1 AND S2 |
| S2 | “health science*” or medicine or medical or anaesthesiologist* or anesthesiologist* or dentist* or dental* or doctor* or physician* or "nurse practitioner*" or nurse or clinician* or midwi* or pharmacist* or physiotherap* or "occupational therap*" or radiographer* or "medical personnel" or "medical professional*" or "medical worker*" or "medical provider*" or "medical staff" or "health personnel" or "healthcare personnel" or "health professional*" or "health care professional*" or "healthcare professional*" or "health worker*" or "health care worker*" or "healthcare worker*" or "health provider*" or "health care provider*" or "healthcare provider*" or "health staff" or "health care staff" or "healthcare staff" or Paramedic* or "health manager*" or "health care manager*" or "healthcare manager*" or "clinical officer*" or "community health aid*" or “Physical therap*” or “rehabilitation therapy” or osteopath* |
| S1 | “quality improvement” or “self assessment*” or metacognition or “scientific method” or “problem solving” or “critical judgment” or “critical thinking” |
